# 老年弥漫大B细胞淋巴瘤诊断与治疗中国专家共识（2024年版）

**DOI:** 10.3760/cma.j.cn121090-20231228-00343

**Published:** 2024-04

**Authors:** 中华医学会血液学分会淋巴细胞疾病学组 中国临床肿瘤学会（CSCO）淋巴瘤专家委员会

**Keywords:** 淋巴瘤，大B细胞，弥漫性, 老年, 诊断, 治疗, Lymphoma, large B-cell, diffuse, Geriatric, Diagnosis, Therapy

## Abstract

随着人口老龄化来临，老年弥漫大B细胞淋巴瘤（Diffuse large B cell lymphoma，DLBCL）患者比例上升。老年患者对治疗耐受性差，治疗方案需要个体化制定。为提高我国临床医师对老年DLBCL的诊断及治疗水平，中华医学会血液病学分会淋巴细胞疾病学组和中国临床肿瘤学会（CSCO）淋巴瘤专家委员会组织相关专家制定了本共识。

## Abstract (en)

The general population in China is aging, and thus the number of older patients with diffuse large B-cell lymphoma (DLBCL) will continue to increase. Individualized treatment is required to maximize therapeutic potential while minimizing the risk of toxicity. To improve the diagnosis and treatment of DLBCL in older people in China, the Lymphocyte Disease Group of the Hematology Division of the Chinese Medical Association and Lymphoma Expert Commitee of Chinese Society of Clinical Oncology (CSCO) have organized relevant experts to formulate this consensus.

弥漫大B细胞淋巴瘤（Diffuse large B cell lymphoma，DLBCL）是最常见的恶性淋巴瘤亚型，国内数据显示中位诊断年龄约57岁[1]，近30％的患者诊断时年龄超过70岁，且发病率随年龄的增加而上升[2]。根据中国社会保障协会公布的《中国老龄化研究报告2022》，2020年我国65岁以上老龄人口达到1.91亿人，占总人口比例为13.5％，预计2057年65岁以上人口达4.25亿人的峰值，占总人口比例32.9％～37.6％[3]。据此估计，我国老年DLBCL患者未来占比将会逐渐增加，亟需重点关注。尽管20多年来以利妥昔单抗（R）为基石的R-CHOP（利妥昔单抗+环磷酰胺+多柔比星+长春新碱+泼尼松）方案显著改善DLBCL患者的预后，但该方案对于老年患者的疗效仍不尽如人意。中国医学科学院肿瘤医院回顾2006至2012年349例接受治疗的老年DLBCL患者的生存状况，5年无进展生存（PFS）及总生存（OS）率分别为45.8％和51.9％[4]。而同期另一项回顾研究显示，年龄<60岁患者5年PFS和OS率分别为65.4％和74.8％，显著优于老年患者[2]。上海交通大学医学院附属瑞金医院的一项回顾研究也同样证实，即使初诊接受标准免疫化疗患者，PFS和OS率随着年龄增长也呈下降趋势，年龄<60岁患者3年PFS和OS率分别为72.1％和83.2％，而>60岁患者分别为60.8％和71.0％；75岁以上患者则为51.2％和57.6％[5]。此外，因年龄、合并症等因素，临床研究通常将这部分患者排除在外，故缺乏标准的治疗方案[6]。

为规范我国临床医师对老年DLBCL患者的诊治水平，中华医学会血液病学分会淋巴细胞疾病学组和中国临床肿瘤学会（CSCO）相关专家根据国际上相关指南[7]–[8]及循证医学证据，讨论并制定本共识。由于DLBCL各亚型的预后及诊疗存在差异，本共识所指疾病亚型为DLBCL非特指型。

一、老年患者的定义

因不同地区人均寿命差异，目前“老年”的年龄界限尚无统一。研究表明肿瘤患者的数字年龄并不能准确预测治疗的益处，而生物学年龄才更适合评估对治疗强度的耐受性。结合既往临床研究的资料，本共识将年龄≥60岁患者认定为老年DLBCL患者。

二、老年DLBCL患者宿主因素评估

老年综合评估（Comprehensive geriatric assessment，CGA）是从老年学角度出发，根据患者年龄、功能状态、合并症、认知水平、精神状态及营养状态，通过定量评分量表来对患者进行全面评估。CGA可有效预测老年DLBCL患者治疗反应、疾病进展和（或）复发以及远期生存[9]，而且有助于治疗决策，提高生存率[10]。因此，老年DLBCL治疗前进行宿主因素评估具有非常重要的意义。

CGA常用的功能评估量表有日常生活活动能力（Activities of daily living，ADL）、工具性日常生活活动能力（Instrumental activities of daily living，IADL）及评估合并症的量表Charlson指数（CCI）或改良老年疾病累计评分表（MCIRS-G）。ADL评估洗澡、穿衣、如厕、移动、进食和大小便控制等能力，每项得分1分，总分6分；IADL评估财务管理、使用交通工具、采购、日常家务、沟通和服用药物等能力，每项1分，总分8分。目前较成熟的CGA体系有：①意大利淋巴瘤基金会（Lymphoma Italian Foundation，FIL）提出的老年综合评估（FIL-CGA），该体系以年龄、ADL、IADL和MCIRS-G为评估参数，将老年患者分为适合化疗组，不适合化疗组和脆弱组[11]；2021年其更新了评估标准，提出简化CGA体系（sGA）[12]。②日本淋巴瘤治疗学会（Society of lymphoma treatment in Japan）提出ACA指数（Age, Comorbidities, and Albumin index），该体系以年龄、血清白蛋白、CCI为评估参数，年龄≥75岁、血清白蛋白<0.37 g/L、CCI≥3三个指标各积1分，将老年患者分为预后良好（0分）、预后较好（1分）、预后中等（2分）、预后差（3分）4个亚组[13]。③北京医院2018年对ACA指数进行了优化，将IADL加入评估体系，提出IACA指数，评价指标包括：年龄、IADL、CCI、血清白蛋白[14]。④其他：老年评估8项（G8）[15]、虚弱老年人筛查（VES-13）[16]等，国内应用资料较少。目前尚无随机对照研究证实何种评估体系更适合老年DLBCL患者评估及指导分层治疗，建议开展相关研究。国内常用的CGA体系为：FIL-CGA、sGA和IACA指数，具体评分标准见表1～3。

**表1:** 意大利淋巴瘤基金会提出的老年综合评估（GA）的分组标准

指标	CGA分层		
	适合化疗组	不适合化疗组	脆弱组
ADL（分）	6	5	<5
IADL（分）	8^a^	6～7^b^	<6^b^
MCIRS-G	无3～4级合并症（且2级合并症<5个）^a^	无3～4级合并症（且2级合并症5～8个）^b^	≥1个3～4级合并症（或2级合并症>8个）^b^
年龄	<80岁^a^	≥80岁的适合化疗患者^a^	≥80岁的不适合化疗患者^a^

**注** ADL：日常生活活动能力；IADL：工具性日常生活活动能力；MCIRS-G：改良老年疾病累计评分表；^a^ 同时满足；^b^ 或满足

**表2:** 简化老年综合评估体系（sGA）的分组标准

指标	sGA分层			
	适合化疗组	不适合化疗组①	不适合化疗组②	脆弱组
ADL（分）	≥5	<5	6	<6
IADL（分）	≥6	<6	8	<8
MCIRS-G	无3～4级的合并症（2级合并症≤8个）	≥1个3～4级合并症（2级合并症>8个）	无3～4级合并症（2级合并症<5个）	≥1个3～4级合并症（2级的合并症≥5个）
年龄（岁）	<80	<80	≥80	≥80

**注** ADL：日常生活活动能力；IADL：工具性日常生活活动能力；MCIRS-G：改良老年疾病累计评分表

**表3:** IACA指数评估系统分组标准

指标	IACA评分（分）		
	0	1	2
IADL	8	6～7	≤5
血清白蛋白（g/L）	≥34	<34	–
CCI	<3	≥3	–
年龄（岁）	≤75	>75	–

**注** IACA指数：纳入IADL的年龄-合并症-白蛋白指数；IADL：工具性日常生活活动能力；CCI：Charlson指数；–:无内容；计算4项参数得分，总分得0分为适合组，1～2分为不适合组，≥3分为脆弱组

三、诊断及鉴别诊断、危险度评估

老年DLBCL诊断和鉴别诊断参考国家卫生健康委员会发布的《弥漫性大B细胞淋巴瘤诊疗指南（2022年版）》。需要重点指出，老年DLBCL患者基因组变异更加复杂，包括MYC、BCL2表达显著上升，不良预后基因改变增多[17]，且随着年龄增长，活化B细胞样（ABC）亚型比例上升[18]。同时，老年患者肿瘤微环境也存在自身的特点，如效应免疫细胞功能减弱等[5],[19]。上述因素是导致老年患者预后较差的内在原因。因此，建议老年DLBCL患者尽可能行二代测序和肿瘤免疫微环境等分子学检查，有助于早期判断预后及开展精准治疗研究。

老年DLBCL预后评估建议采用老年预后指数模型，其纳入指标包括：sGA，淋巴瘤国际预后评分（IPI）和HGB，将患者分为低、中、高危，预测3年生存率分别为87％，69％和42％[12]，具体评分标准见表4。

**表4:** 老年预后指数模型评估系统

指标	评分（分）
sGA	
适合化疗	–
不适合化疗	3
脆弱	4
IPI	
1分	–
2分	1
3～5分	3
HGB<120 g/L	1

**注** sGA：简易老年评估体系；IPI：淋巴瘤国际预后评分；–:不积分；计算3项参数得分，总分得0～1分为低危组，2～5分为中危组，6～8分为高危组

四、老年DLBCL患者治疗原则

目前，老年DLBCL患者尚无标准的一线治疗，优先推荐进入临床研究。部分患者接受R-CHOP样化疗可以达到治愈的目的，但治疗决策前需充分考虑疗效与安全性的平衡，强调动态使用CGA评估体系。需要特别指出的是部分脆弱患者经“预治疗”或支持治疗后，脏器功能得以改善，给予恰当治疗，可获得最佳疗效[14]。老年患者更需重视支持治疗，包括营养支持、感染预防、粒细胞集落刺激因子的使用等。一线治疗流程见图1。

**图1:**
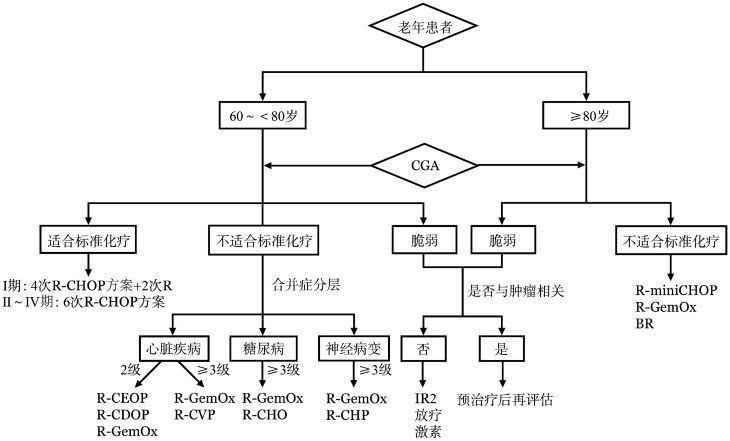
老年弥漫大B细胞淋巴瘤患者一线治疗流程图 **注** CGA：老年综合评估；R-CHOP：利妥昔单抗+环磷酰胺+多柔比星+长春新碱+泼尼松；R-CEOP：利妥昔单抗+环磷酰胺+依托泊苷+长春新碱+泼尼松；R-CDOP：利妥昔单抗+环磷酰胺+脂质体多柔比星+长春新碱+泼尼松；R-CVP：利妥昔单抗+环磷酰胺+长春新碱+泼尼松；R-CHO：利妥昔单抗+环磷酰胺+多柔比星+长春新碱；R-CHP：利妥昔单抗+环磷酰胺+多柔比星+泼尼松；R-GemOx：利妥昔单抗+吉西他滨+奥沙利铂；R-miniCHOP：利妥昔单抗+减剂量的CHOP；BR：利妥昔单抗+苯达莫司汀；IR2：伊布替尼+利妥昔单抗+来那度胺

1. 60～<80岁且适合标准化疗，治疗以根治为目的：法国成人淋巴瘤协作组（GELA）开展的LNH98-5研究，入组初诊年龄介于60～80岁，疾病分期Ⅱ～Ⅳ期且无中枢神经系统累及，并且体能状态（PS）良好（PS≤2分）的患者，给予8次R-CHOP方案治疗，总有效率（ORR）为75.0％，5年PFS率为54.0％，5年OS率为58.0％，治疗相关死亡风险为6.0％[20]；10年PFS和OS率分别为36.5％和43.5％，诊断5年后复发风险为7.0％[21]。后期研究进一步显示6次R-CHOP方案疗效与8次相当[22]。基于这两项研究，本共识将6次R-CHOP方案作为60～<80岁适合强治疗患者的优先选择。对于疾病分期Ⅰ期且无大包块的老年患者，上海交通大学医学院附属瑞金医院的结果表明4次R-CHOP方案后获得完全代谢缓解（CMR）患者，序贯单纯4次R与其联合2次CHOP方案的疗效相当，有望进一步降低化疗相关不良反应[23]。

基于全球多中心、双盲、随机对照的Ⅲ期POLARIX临床试验结果[24]，维泊妥珠单抗（pola）国内已获批联合R-CHP（利妥昔单抗+环磷酰胺+多柔比星+泼尼松）用于既往未经治疗的DLBCL成人患者。探索性亚组分析显示，60岁以上患者应用pola-R-CHP方案较R-CHOP方案可能有更好地改善PFS趋势，2年PFS率分别为77.9％和69.5％；主要不良反应为周围神经病变及恶心等。

2. 60岁以上且不适合标准化疗，治疗尽可能以根治为目的：>60岁老年人的慢性病共病患病率为43.6％[25]，R-CHOP化疗导致的心脏事件发生率近30.0％[26]。早期一项前瞻性研究在R-CHOP基础上根据CGA体系进行药物及剂量调整，不适合标准化疗组5年OS率低于标准化疗组（53.0％对76.0％）[10]。为降低蒽环类药物的心脏不良反应，也可采用脂质体多柔比星替代多柔比星[27]。提高治疗耐受性的另一策略为更换一线治疗方案。江苏省人民医院单中心前瞻性研究显示，年龄>70岁（PS>2分占28％），或年龄在60～70岁且PS>2分但无严重合并症的患者，接受R-GemOx（利妥昔单抗+吉西他滨+奥沙利铂）方案治疗，ORR为75％，3年PFS和OS率分别为49.0％和65.0％，主要不良反应是骨髓抑制及胃肠道症状[28]。综合疗效及安全性特点，本共识对于老年不适合强治疗患者建议根据CGA个体化选择R-CHOP样或R-GemOx方案。

年龄≥80岁，体能状态良好（PS≤2分）且无严重合并症的患者，给予6次R-miniCHOP（利妥昔单抗+减剂量的CHOP）方案治疗，ORR为73.0％，2年PFS和OS率分别为47.0％和59.0％，治疗相关死亡风险8.0％[29]，优先推荐。R-miniCHOP方案基础上联合免疫调节剂并未带来生存获益[30]，本共识不作常规推荐。华中科技大学同济医学院附属协和医院一项小样本前瞻性对照研究纳入年龄为70～82岁，经CGA评估为不适合强治疗初治患者，对比BR（苯达莫司汀+利妥昔单抗）与R-miniCHOP方案的疗效，结果显示两者相当[31]。因此，对于不耐受R-miniCHOP方案治疗的患者，BR方案也可做备选。

3. 60岁以上脆弱患者，治疗以症状改善为目的，但亦可获得长期缓解：研究显示，脆弱患者接受以根治为目的的免疫化疗，1年内死亡风险超过50.0％，但中位生存时间近3.5年[32]，因此，对这部分患者治疗更强调个体化原则。如脆弱因肿瘤引起（比如3个月内体能状态急剧下降等），建议通过“预治疗”模式，如激素、环磷酰胺和泼尼松等方案，动态行CGA评估，制定恰当的个体化治疗方案[33]。近年随着抗体类药物、小分子化合物等在血液肿瘤取得良好临床效果，无化疗方案成为老年脆弱患者治疗探索的方向。上海交通大学医学院附属瑞金医院开展的一项单中心Ⅱ期研究入组30例中位年龄80（76～92）岁，经CGA评估为不适合强治疗/脆弱初诊DLBCL患者（其中脆弱患者比例为80％），评估IR2（伊布替尼+来那度胺+利妥昔单抗）方案疗效及安全性，中位随访时间为27.6个月，2年PFS和OS率分别为53.3％和66.7％，亚组显示IPI评分为低危或低中危患者疗效更明显，2年PFS和OS率分别为88.9％和100％；主要不良反应是骨髓抑制、肺部感染及房颤等[34]。因此对于60岁以上脆弱患者，布鲁顿酪氨酸激酶（BTK）抑制剂联合来那度胺、R也可做备选。

五、老年DLBCL患者维持治疗的问题

探索维持治疗主要针对获得部分缓解（PR）且不适合强化疗的老年患者。尽管REMARC研究显示R-CHOP方案治疗后达到完全缓解（CR）或PR的老年DLBCL患者接受来那度胺维持治疗2年，2年PFS率较安慰剂组提高（从75.0％提升到80.0％），但未转化为OS获益[35]。维持治疗能否获益及哪些患者能获益依然不明确[36]，本共识暂不作为常规推荐，由主管医师根据患者具体情况进行选择。

六、难治/复发老年DLBCL患者的治疗

难治DLBCL的定义为符合下述标准之一：①经一线免疫化疗4个疗程或二线及以上免疫化疗2个疗程后，最佳疗效未获得PR及以上疗效；②auto-HSCT后12个月内复发[37]–[38]。复发DLBCL的定义为获得CR后再次出现新发病灶，其中自接受治疗起2年内出现为早期复发，2年后为晚期复发[39]。上述定义参考年轻患者，其在老年患者中的临床意义仍有待进一步明确。

上海交通大学医学院附属瑞金医院牵头全国多中心开展的一项回顾性研究（REAL-TREND研究），调研经一线接受免疫化疗后难治患者的生存状况，入组2 342例患者（>60岁占38.6％），难治发生比例为14.9％（350/2 342），其中一线治疗后难治比例为51.7％（181/350）；一线治疗后复发/进展比例为20.3％（438/2 161），移植后12个月内复发比例为3.7％（16/438）[37]。难治/复发患者的预后差异较大[37],[40]，复发时IPI评分为4～5分、中枢神经系统复发及挽救治疗未获得PR以上疗效均与不良预后相关[37]。国内真实世界难治/复发老年DLBCL患者的生存状况亟待调研。

尽管关于CGA评分临床应用的数据主要来自初始治疗，但本共识仍推荐老年难治/复发DLBCL患者治疗前进行CGA评分，有助于临床决策。

以下仅列举部分证据相对充分的难治/复发老年DLBCL治疗方案，但不限于此：

1. auto-HSCT是挽救治疗敏感DLBCL患者争取再次治愈机会的有效治疗手段，是当前标准治疗选择[41]。挽救治疗获得CR患者接受auto-HSCT，中位OS期为50.4个月；获得PR患者，中位OS期仅为12.2个月[37]。但随年龄增长，治疗相关不良反应也随之增加[42]。因此，对于年龄<75岁患者，充分评估治疗风险及生存获益后可选择接受auto-HSCT。

2. 嵌合抗原受体T（CAR-T）细胞治疗是治疗难治/复发B细胞淋巴瘤的新疗法，目前获批适应证包括：①一线免疫化疗无效或在一线免疫化疗后12个月内复发的DLBCL；②二线或以上系统性治疗后复发或难治性DLBCL。CAR-T细胞疗效，与年龄、脏器功能不全等无相关性，但PS>1分患者PFS和OS期均显著缩短[43]–[44]。ZUMA-7研究入组51例年龄≥65岁难治DLBCL患者，CR率高于对照组（75.0％对33.0％），2年无事件生存（EFS）率高于对照组（47.8％对15.1％）；3级以上不良反应发生比例与对照组相似[45]。本共识对于美国东部肿瘤协作组（ECOG）评分<2分，或经过桥接治疗体能状况改善的难治/复发老年DLBCL患者推荐行CAR-T细胞治疗。

3. pola-BR方案已获批用于不适合接受造血干细胞移植的难治/复发 DLBCL成人患者。pola-BR方案用于治疗难治/复发DLBCL是基于一项多中心、开放标签的Ⅰb～Ⅱ期试验，纳入80例不适合auto-HSCT的难治/复发DLBCL患者（pola-BR方案40例，BR方案40例），接受pola-BR方案组治疗结束时的CR率为42.5％，中位缓解持续时间（DoR）为10.9个月；中位PFS和OS期分别为9.2个月和12.4个月，但需注意治疗相关不良反应，如骨髓抑制、感染及周围神经病变等[46]–[47]。合并周围神经病变的患者需避免使用。

4. 格菲妥单抗（glofitamab）获批用于治疗既往接受过至少二线系统性治疗的难治/复发DLBCL成人患者。一项Ⅰ/Ⅱ期剂量递增研究评估奥妥珠单抗预处理联合格菲妥单抗治疗难治/复发DLBCL成人患者的疗效与安全性，入组154例（110例为DLBCL非特指型）均接受Ⅱ期推荐剂量，≥65岁患者占54.5％；59.7％的患者接受过二线以上治疗，33.1％的患者接受过CAR-T细胞治疗；CR率为39.0％，中位随访12.6个月，中位DoR未达到，1年PFS和OS率分别为37.0％和50.0％；亚组分析显示，65岁以上患者获益与总体相当。该药治疗相关不良反应发生率较高，以细胞因子释放综合征和神经毒性最为常见，但多数不良反应为1～2级[48]。

5. 对于脆弱且多线复发的患者，局部放疗、BTK抑制剂、免疫调节剂等单药或联合均可尝试使用，以改善症状为主[49]。

七、疗效评价及随访

参考国家卫生健康委员会发布的《弥漫性大B细胞淋巴瘤诊疗指南（2022年版）》。
